# CircRNA TADA2A relieves idiopathic pulmonary fibrosis by inhibiting proliferation and activation of fibroblasts

**DOI:** 10.1038/s41419-020-02747-9

**Published:** 2020-07-21

**Authors:** Juan Li, Ping Li, Guojun Zhang, Pan Qin, Da Zhang, Wei Zhao

**Affiliations:** 1 Department of Respiratory Medicine, The First Affiliated Hospital of Zhengzhou University, 450052 Zhengzhou, China; 2 Department of Pediatric Surgery, The First Affiliated Hospital of Zhengzhou University, 450052 Zhengzhou, China

**Keywords:** Biochemistry, Cell biology

## Abstract

The excessive activation and proliferation of lung fibroblasts are responsible for the abundant deposition of extracellular matrix (ECM) in idiopathic pulmonary fibrosis (IPF), while its specific mechanism is still unknown. This study focuses on the role of circRNA (circ) TADA2A in functional abnormalities of lung fibroblasts and aims to elaborate its regulatory mechanism. In the present study, circTADA2A was downregulated in both IPF primary human lung fibroblasts and human IPF fibroblastic cell lines. Functionally, the overexpression of circTADA2A repressed the activation and proliferation of normal human fibroblastic cell line induced by several fibrogenic growth factors. Using fluorescence in situ hybridization (FISH), luciferase reporter assays, and RNA pull-down, circTADA2A was confirmed to function as sponges of miR-526b and miR-203, thus releasing the expression of Caveolin (Cav)-1 and Cav2. The overexpression of circTADA2A suppressed lung-fibroblasts activation via Cav1 and reduced lung-fibroblasts proliferation via Cav2. In vivo experiments also confirmed that the overexpression of circTADA2A decreased fibrogenic responses induced by bleomycin in lung-fibrosis mice. Collectively, circTADA2A repressed lung-fibroblasts activation via miR-526b/Cav1 and reduced lung-fibroblasts proliferation via miR-203/Cav2, thus inhibiting the excessive deposition of ECM and relieving IPF.

## Introduction

Idiopathic pulmonary fibrosis (IPF) is the most common form of interstitial lung disease, with high morbidity and lacking medical therapies to improve the survival rate. The representative characteristic of IPF is the abundant deposition of extracellular matrix (ECM) in the alveolar parenchyma, which will sequentially irreversible alters normal lung architecture and ultimately resulting in respiratory failure^1^. As a major producer of ECM, the significant role of activated fibroblasts in the development of IPF is apparent.

Fibroblasts serve a crucial role in tissue remodeling and fibrosis. In normal wound healing, activated fibroblasts secrete ECM to provide a scaffold for the restoration of normal lung architecture and eventually undergo apoptosis to restrain the excessive production of ECM^2^. Nevertheless, in the lung tissues of patients with IPF, fibroblasts present several dysfunctions, including the apoptosis resistance, inundant proliferation, and sustained activation^3^, which lead to the massive accumulation of ECM and given rise to fibrosis. Although the mechanism of the excessive activation and proliferation of fibroblasts has not been expounded explicitly, several fibrogenic growth factors [platelet-derived growth factor (PDGF), insulin-like growth factor 1 (IGF-1), and transforming growth factor-β1 (TGF-β1)] have been proved to contribute to this process during the development of IPF^4^. If the functional abnormalities of fibroblasts, which located in or around pulmonary fiber lesion can be restrained, it will be of great significance to the clinical treatment of IPF.

Circular RNA (circRNA) is a new class of endogenous noncoding RNA, generating in the back-splicing process of pre-mRNA. The majority of circRNAs are highly conserved across species and play regulatory roles in the onset and progression of numerous human diseases. A recent study revealed that several circRNAs were dysregulated in the plasma of IPF patients^5^, indicating circRNAs may play a potential role in the development of IPF. Besides, emerging evidence showed that circRNAs owned the ability to regulate fibroblasts proliferation and activation via acting as the sponges of microRNAs (miRNAs), binding to them directly and removing their suppressive effect on mRNA expression^6^. Zhu et al.^7^ found that the overexpression of circNFIB notably decreased the proliferation of primary mice cardiac fibroblasts via sponging miR-433. Tang et al.^8^ reported circRNA_000203 exhibited its promotion effect on the activation of mice cardiac fibroblasts through targeting miR-26b-5p. Inspired by these findings, we speculated that circRNA could also function as miRNA sponges, regulate proliferation and activation of lung fibroblasts, thus influencing the development of IPF.

## Materials and methods

### Isolation and growth of human primary lung fibroblasts

IPF primary human lung fibroblasts (IPF-HLF) were acquired from diagnostic biopsies of IPF patients (*n* = 5) and normal primary human lung fibroblasts (N-HLF) were acquired from healthy donor lung tissues failed for transplantation (*n* = 5). The diagnosis of IPF was supported by history, physical examination, pulmonary function tests, and typical high-resolution chest computed tomography findings of IPF. The IPF patients (*n* = 5) and healthy donors (*n* = 5) were all male and aged from 40 to 65 years. Exclusion criteria included current or recent use of immunosuppression; chronic infection such as HIV or hepatitis; known pulmonary hypertension; cardiovascular, renal, or neoplastic disease; and inability to provide informed consent. This study was approved by the Ethics Committees of The First Affiliated Hospital of Zhengzhou University and written informed consent was obtained on all patients prior to the procedure being performed.

The human primary lung fibroblasts were isolated as previously described^9^. Briefly, lung tissues were cut into small pieces and subjected to enzymatic dissociation in Hank’s balanced salt solution (containing 600 U/ml collagenase I, 2 U/ml papain, 2 U/ml protease, and 3.8 mM calcium chloride) at 37 °C for 1 h. Next, tissues were ground by glass pipette trituration, and the supernatant was centrifuged at 800 × *g* for 5 min to collect the cells. Then, cells were redispersed in high-glucose Dulbecco’s Modified Eagle Medium (DMEM) containing 10% fetal bovine serum (FBS), 50 U/ml strepto-ECM, and 50 μg/ml penicillin. The phenotype of human primary lung fibroblasts was confirmed by positive immunocytochemistry for vimentin (data not shown). In all experiments, the lung fibroblasts were harvest in passages four to six for the test.

### Cell culture

The human IPF fibroblastic cell lines (LL-97A and LL-29) and normal human fibroblastic cell line (LL-24) were obtained from the American Type Culture Collection (USA), authenticated and tested for mycoplasma contamination from the Procell (Chnia). Cells were maintained in Kaighn’s Modification of Ham’s F-12 Medium (F-12K Medium) containing 15% FBS and cultured with 5% CO_2_ at 37 °C. The hyperproliferation and activation of normal human lung fibroblasts were performed as previously described^4^. Briefly, LL-24 cells were starved for 48 h and incubated with fetal calf serum (FCS; 2% or 5%), PDGF-BB (30 or 60 ng/ml), and IGF-1 (100 or 200 ng/ml) for 6 h to promote fibroblasts proliferation and incubated with TGF-β1 (5 or 10 ng/ml) for 6 h to activate fibroblasts. All growth factors and cytokines were purchased from Solarbio (China).

### Cell transfection and infection

Cells were cultured in 6-well plates with a concentration of 4 × 10^5^ cells/well. When the cells were cultured to 70% confluence, cells were transfected with RNAi-vector [si-circTADA2A, si- Caveolin-1 (Cav1), and si-Cav2] or microRNA inhibitors (miR-526b inhibitor and miR-203 inhibitor) or microRNA mimics (miR-526b mimic and miR-203 mimic) or their relative negative controls (si-control, NC, and Pre-NC) using Lipofectamine 2000 (Invitrogen, USA). Cells were incubated with 2 ml Opti-MEM medium (GIBCO, USA) containing plasmids (1 μg) and Lipofectamine 3000 (2.5 μl). The medium was changed after 6 h, and the RNA extraction was performed at 48 h to verify the transfection efficiency.

To overexpress circTADA2A or Cav1 or Cav2, the adenovirus-expressed circTADA2A or Cav1 or Cav2 (Ad-circTADA2A or Ad-Cav1 or Ad-Cav2) were produced by Ribobio (China). The appropriate volume of virus particles calculated by the multiplicity of infection (MOI) was added in the cell-culture medium. Forty-eight hours later, virus infection efficiency was monitored by GFP expression using the fluorescence microscope. Ad-GFP was used as a negative control. The sequences of the transfected components were shown in Table 1.

**Table 1:** Sequences of the transfected components or primers used in the experiments.

Transfected components	Sequences
si-circTADA2A	5′-ATTCCATTTCACTACTTCAGA-3′
si-Cav1	5′-GCAAAUACGUAAUGUACAAGU-3′
si-Cav2	5′-GGAGAUUGGGAUACUGUAAUA-3′
miR-526b mimic	5′-CTCTTGAGGGAAGCACTTTCTGT-3′
miR-203 mimic	5′-GUGAAAUGUUUAGGACCACUAG -3′
miR-526b inhibitor	5′-ACAGAAAGTGCTTCCCTCAAGAG-3′
miR-203 inhibitor	5′-CUAGUGGUCCUAAACAUUUCAC-3′
Ad-circTADA2A (for circTADA2A amplification)	F: 5′-CAAGGAGGAGTGTGAGAAGCACT-3’
	R: 5′-GTCTTGGTGCACATTTGATTGGCT-3′

Primers	Sequences
circANKRD42	F: 5′‑CTGGACAAGGCCACATAGAGT‑3′
	R: 5′‑CAGAGCAGCCAATGAAGACAC‑3′
circCDC27	F: 5′‑TCTATTAGGGCATGAGTTTGTCTT‑3′
	R: 5′‑TCCTTGGTTGTGGAGCTGTC‑3′
circZMYM2	F: 5′‑GCACCTGACAGCATCTATTACC‑3′
	R: 5′‑GACAGTAGAAACGCAGTAAGCAA‑3′
circGRHPR	F: 5′‑ACAGATACCACCGCCGAACT‑3′
	R: 5′‑TCTAGCTCCTTGGCAGGGAT‑3′
circTADA2A	F: 5′‑AGCCATTCCATTTCACTACT‑3′
	R: 5′‑CCACAGTCCATCACAGCTTC‑3′
circARHGAP26	F: 5′‑CCATGCAAGCTTTGTCGGAA‑3′
	R: 5′‑CATACTTCTTTTTGGCTTCA‑3′
miR-203	F: 5′-GGGGTGAAATGTTTAGGAC-3′
	R: 5′-CAGTGCGTGTCGTGGAGT-3′
miR-520f	F: 5′-ACACTCCAGCTGGGAAGTGCTTCCTTTTAG-3′
	R: 5′-CTCAACTGGTGTCGTGGAGTCGGCAATTCAGTTGAGAACCCTCT-3′
miR-450b-3p	F: 5′-GATCCCCGGAUGCAAAAUGAUCCCAATTCA-3′
	R: 5′-AGCTTAAAAAGGAUGCAAA AUGAUCCCAAT-3′
miR-526b	F: 5′-GTCTCTTGAGGGAAGCACT-3′
	R: 5′-GTGCAGGGTCCGAGGT-3′
miR-638	F: 5′-AGGGATCGCGGGCGGGTGGCGGCCT-3′
	R: 5′-ATTCTAGAGGCCGAGGCGGCCGACA TGT-3′
miR-769-3p	F: 5′-TCGGCAGGCTGGGATCTCCGGGG-3′
	R: 5′-GTGCAGGGTCCGAGGT-3′
U6	F: 5′-CTCGCTTCGGCAGCACA-3′
	R: 5′-AACGCTTCACGAATTTGCGT-3′
GAPDH	F: 5′-TCACTCAAGATTGTCAGCAA-3′
	R: 5′-AGATCCACGACGGACACATT-3′
LUC-circTADA2A vector	F: 5′-CCGCTCGAGGCAGGATGTAGCCAATCAAAT-3′
	R: 5′-ATAAGAATGCGGCCGCAGTGAAATGGAATGGCTGTGT-3′
circTADA2A probe	5′-CATCCTGCAGTGAAATGGAATGGC-3′

### Cell proliferation assay

Lung-fibroblasts proliferation was detected by BrdU incorporation assay. Cells were cultured in 96-well plates with a concentration of 3000 cells/well. When the cells reached confluence, cells were incubated with BrdU for four hours. After that, the BrdU incorporation was measured using an ELISA BrdU assay kit (Abcam, UK).

### Quantitative RT-PCR

Total RNAs were isolated from cells and lung tissues of lung-fibrosis mice using TRIzol Reagent (Invitrogen, USA). The quality of total RNA samples was evaluated by spectrophotometer and the high-quality RNAs (1.8 < OD260/280 < 2.0) were inversely transcribed into cDNA using a cDNA synthesis kit (ThermoFisher, USA). Quantitative RT-PCR was performed to measure circRNAs and miRNAs expressions using the Thunderbird SYBR qPCR mix (Toyobo, Japan). Gene expressions were calculated by the 2^−∆∆CT^ method, the relative expressions of circRNA and miRNA were normalized to GAPDH and U6 respectively. The sequences of qRT-PCR primers were shown in Table 1.

### Western blot

The determination of protein levels of collagen 1a1 (COL1A1), collagen 3a1 (COL3A1), α-smooth muscle actin (α-SMA), laminin (LN), fibronectin (FN), Caveolin-1 (Cav1), and Caveolin-2 (Cav2) were done by western blot with total protein purified from cell lysate or lung tissues of lung-fibrosis mice by RIPA lysis buffer. Proteins were subjected to 10% sodium dodecyl sulfate-polyacrylamide gel electrophoresis and then transferred to PDVF membrane (ThermoFisher Scientific, USA). After being blocked with 5% skim milk for 30 min, membranes were incubated with primary antibodies (against COL1A1, COL3A1, α-SMA, LN, FN, Cav1, and Cav2). After the night, the membranes were incubated with the secondary antibodies specifically. Immunoblots were visualized in IBright FL1500 Intelligent Imaging System (ThermoFisher, USA) and GAPDH was used as an internal control.

The primary antibodies used in the experiment were as follows: anti-COL1A1 (1:1000; sc-59772, Santa Cruz Biotechnology, USA), anti-COL3A1 (1:1000; sc-271249, Santa Cruz Biotechnology, USA), anti-α-SMA (1:1000; ab32575, Abcam, UK), anti-LN (1:2000; ab11575, Abcam, UK), anti-FN (1:1000; ab268021, Abcam, UK), anti-Cav1 (1 µg/ml; ab2910, Abcam, UK), anti-Cav2 (1:5000; ab133484, Abcam, UK), and GAPDH (1:2500; ab9485, Abcam, UK). The secondary antibody used in the experiment were m-IgGκ BP-HRP (1:10000; sc-516102, Santa Cruz Biotechnology, USA) and Goat Anti-Rabbit IgG H&L (1:5000; ab205718, Abcam, UK).

### Dual-luciferase reporter gene assay

To verify the combination of circTADA2A and miR-526b/miR-203, the sequence of circTADA2A was amplified and inserted into pGL3-basic plasmids. 0.5 μg plasmid and 20 nM miR-526b mimic or miR-203 mimic or mimic-negative control (Pre-NC) were co-transfected in well-grown 293 T cells by using lipofectamine 2000 (ThermoFisher, USA). Forty-eight hours after transfection, the cells were lysed and the activities of Renilla luciferase and firefly luciferase were measured with Dual-luciferase Reporter Assay Kit (Promega, China) following the manufacturer’s protocol. To verify the combination of miR-526b and Cav1/miR-203 and Cav2. The luciferase activities of Cav1-3′-UTR-mutant (Cav1 UTR WT), Cav2-3′-UTR-mutant (Cav2 UTR WT), and Cav2-3′-UTR-mutant (Cav2 UTR WT) were measured in the same way. The primer sequences were shown in Table 1.

### RNA pull-down assay

The combination of circTADA2A and miR-526b/miR-203 was determined by RNA pull-down assay. The circTADA2A probe and its negative control [oligonucleotide probe (Oligo probe)] were constructed by Ribobio (China). LL-29 cells (1.5 × 10^7^) were collected and lysed using 100 μl lysis buffer. The lysate was then incubated with 50 pmol biotin-labeled circTADA2A probe and 50 μl streptavidin agarose magnetic beads for 1 h at 4 °C. miR-526b and miR-203 in circTADA2A probe pull-down complex were detected by qRT-PCR using the Oligo probe pull-down complex as a negative control. The probe sequences were shown in Table 1.

### Fluorescence in situ hybridization (FISH)

LL-29 cells were cultured on the coverslips, fixed with 4% paraformaldehyde for 15 min., incubated with proteinase K, and washed with alcohol solutions. Then, the slides were incubated with hybridization solution for 30 min at 37 °C. Cy3-labeled circTADA2A probe and FAM-labeled miR-526b/miR-203 probes were denatured for 8 min at 73 °C and hybridized to the slides for 24 h at 42 °C. Then, blocking was performed and 4,6-diamidino-2-phenyl-indole (DAPI) was used to counterstain the cell nuclei. Finally, the images were obtained with a confocal microscope (Carl Zeiss, Germany).

### Mouse model of IPF

Male C57BL/6 mice (4–6 weeks old, 18–22 g weight) were purchased from Laboratory Animal Resources, Chinese Academy of Sciences (Beijing, China). The Ad-circTADA2A and its negative control (Ad-vector) were produced by Ribobio (China). Before the establishment of a lung-fibrosis mice model, 50 μl saline containing 1 × 10^11^ virus particles of Ad-circTADA2A or Ad-vector were injected in mice intratracheally. After 2 days of injection, mice were randomly divided into four groups. In bleomycin (BLM; *n* = 7), BLM + vector (*n* = 7), and BLM + circTADA2A (*n* = 7) groups, BLM (3 U/kg) in 50 μl saline was administered intratracheally in mice using trachea cannula. In the saline group (*n* = 7), 50 μl saline was administered intratracheally in mice using the same way. Two weeks later, lung-function measurements (total lung capacity, lung compliance, and tissue resistance) were detected by Resistance and Compliance Plethysmographs (Yuyan Instruments, China), and then mice were sacrificed for the following experiment. The animal chosen and group-dividing were random and the investigator was blinded to the group allocation. All protocols in this study have been approved by the Ethics Committee of The First Affiliated Hospital of Zhengzhou University.

### Lung histological examination

For H&E staining, fresh lung tissues were fixed in 4% PFA, embedded in paraffin, and sectioned using an automatic slicing machine (Leica, Germany). The slices were undergone deparaffinating and rehydration and then stained with hematoxylin and eosin (Nanjing Jiancheng Bioengineering Institute, China).

To evaluate the deposition of collagen in lung tissues, Masson trichrome staining was performed as previously described^10^. Briefly, the slices were orderly stained with hematoxylin, ponceau acid fuchsin, and aniline blue. Finally, images were captured using a microscope (Nikon, Japan).

### Hydroxyproline assay

The assessment of hydroxyproline content in lung tissues was performed using a Hydroxyproline Assay Kit (Abcam, USA) under the manufacturer’s protocol. The absorbances of samples at 550 nm were obtained utilizing a microplate reader. The results were expressed as µg/mg lung tissues.

### Statistical analysis

Each experiment was repeated more than three times. Experimental results were expressed as mean ± standard deviation (SD) and analyzed using GraphPad 7.0 Prism. The differences were analyzed by Student *t*-test or one-way analysis of variance (ANOVA) followed by LSD post-hoc test to compare the differences between two groups or more than two groups, respectively. Results were considered statistically significant when *P* < 0.05.

## Results

### CircTADA2A was downregulated in IPF lung fibroblasts

**Fig. 1:**
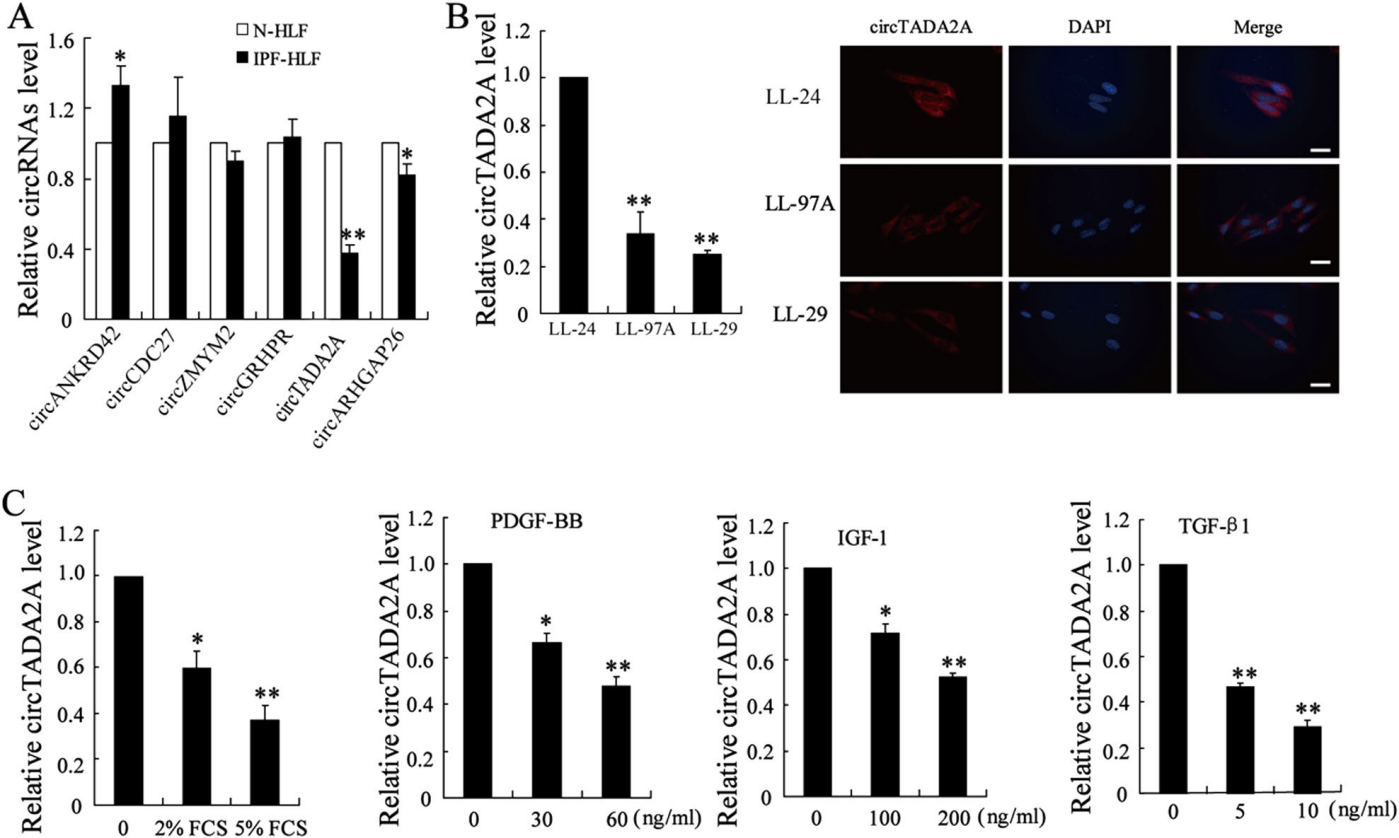
CircTADA2A was downregulated in IPF lung fibroblasts. **a** Circular RNAs (circRNAs) expression levels in IPF primary human lung fibroblasts (IPF-HLF; *n* = 5) and normal primary human lung fibroblasts (N-HLF; *n* = 5) were measured by qRT-PCR. *P < 0.05, **P < 0.01 vs N-HLF. **b** Left: CircTADA2A expression in human IPF fibroblastic cell lines (LL-97A and LL-29) and normal human fibroblastic cell line (LL-24) was measured by qRT-PCR. Right: Representative fluorescence in situ hybridization (FISH) images of circTADA2A in LL-97A, LL-29, and LL-24 (blue, DAPI; red spot, positive staining; Scale bar = 20 µm). **P < 0.01 vs LL-24 cells. **c** LL-24 cells were stimulated with fetal calf serum (FCS; 2% or 5%), platelet-derived growth factor-BB (PDGF-BB; 30 or 60 ng/ml), insulin-like growth factor 1 (IGF-1; 100 or 200 ng/ml), and transforming growth factor-β1 (TGF-β1; 5 or 10 ng/ml) for 6 h, respectively. CircTADA2A expression was measured by qRT-PCR. *P < 0.05, **P < 0.01 vs LL-24 cells without stimulation.

Li et al.^5^ identified 67 circRNAs, which were significantly dysregulated in the blood samples of patients with IPF. Among them, six dysregulated circRNAs with high-absolute fold-change and low *P*-values (circANKRD42, circCDC27, circZMYM2, circGRHPR, circTADA2A, and circARHGAP26) were selected in our study. As shown in Fig. 1a, compared with N-HLF, a significant decrease in circTADA2A expression was observed in IPF-HLF. Then, the human IPF fibroblastic cell lines (LL-97A and LL-29) and normal human fibroblastic cell line (LL-24) were used for further study. The results of qRT-PCR and FISH depicted that circTADA2A was mainly located in the cytoplasm and the abundance of circTADA2A was lower in human IPF fibroblastic cell lines than that of the normal human fibroblastic cell line (Fig. 1b). Interestingly, when we added growth factors and cytokines (FCS, PDGF-BB, IGF-1, and TGF-β1, respectively), which have been reported to driven lung-fibroblasts proliferation and activation in vitro^4^, in the culture medium of LL-24 to stimulate fibroblasts, circTADA2A expression was declined in response to these growth factors and cytokines in a dose-dependent manner (Fig. 1c), implying that with the activation of fibroblasts, the circTADA2A expression was gradually decreased.

### CircTADA2A inhibited growth-factors-driven lung-fibroblasts proliferation and activation

**Fig. 2:**
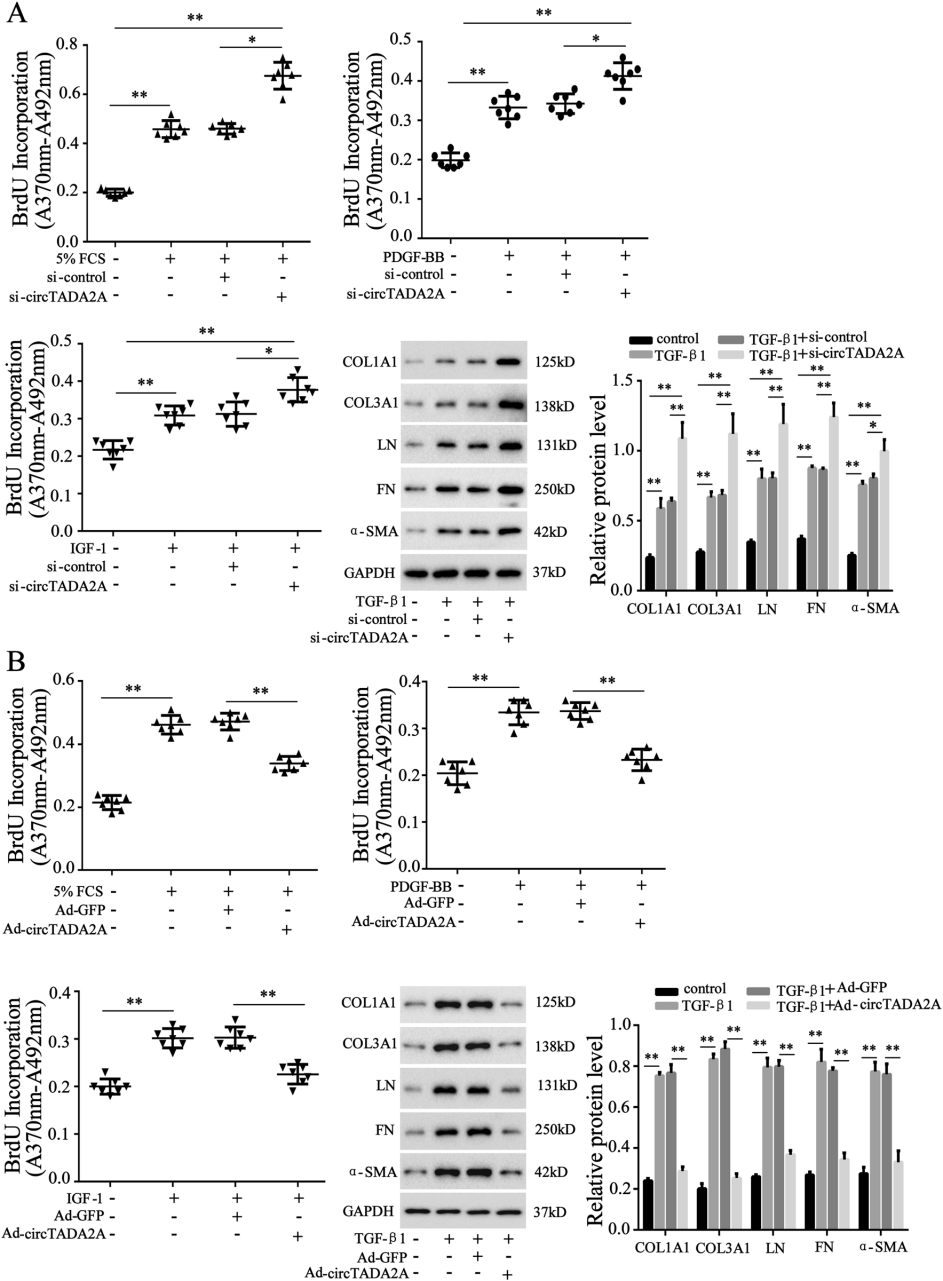
CircTADA2A inhibited growth-factors-driven fibroblasts proliferation and activation. LL-24 cells were transfected with **a** si-circTADA2A or its negative control (si-control) or **b** adenovirus vector (Ad)-circTADA2A or its negative control (Ad-GFP). Cell proliferation was measured by BrdU incorporation assay after stimulation of 5% FCS or 60 ng/ml PDGF-BB or 200 ng/ml IGF-1 for 6 h. The protein expression of collagen 1a1 (COL1A1), collagen 3a1 (COL3A1), laminin (LN), fibronectin (FN), and α-smooth muscle actin (α-SMA) were determined by western blot after stimulation of 10 ng/ml TGF-β1 for 6 h, GAPDH was used as an internal control. *P < 0.05, **P < 0.01.

In light of the above results, the possibility was hypothesized that circTADA2A played a potential role in IPF progression. To verify this hypothesis, si-circTADA2A or Ad-circTADA2A transfection was implemented to silence or overexpress circTADA2A in LL-24 cells. After that, the potential effect of circTADA2A on the proliferation of lung fibroblasts was assessed. The result of the BrdU incorporation assay implied that si-circTADA2A further elevated LL-24 proliferation induced by FCS, PDGF-BB, and IGF-1, respectively, compared with si-control transfection (Fig. 2a). To evaluate the effect of circTADA2A on activation of lung fibroblasts driven by TGF-β1, a potent stimulator of fibroblasts, the protein levels of fibroblasts activation markers [collagen 1a1 (COL1A1), collagen 3a1 (COL3A1), and α-smooth muscle actin (α-SMA)] and components of the extracellular matrix [laminin (LN) and fibronectin (FN)] secreted by activated fibroblasts were determined. As shown in Fig. 2a, the interference of circTADA2A aggravated fibroblasts activation and extracellular matrix secretion. On the contrary, the overexpression of circTADA2A retarded fibroblasts proliferation induced by FCS, PDGF-BB, and IGF-1, and suppressed fibroblasts activated by TGF-β1 (Fig. 2b). Besides, in the human IPF fibroblastic cell line LL-29, the overexpression of circTADA2A reduced the protein levels of COL1A1, COL3A1, α-SMA, LN, and FN (Supplemental Fig. 1).

### CircTADA2A functioned as sponges of miR-203 and miR-526b

**Fig. 3:**
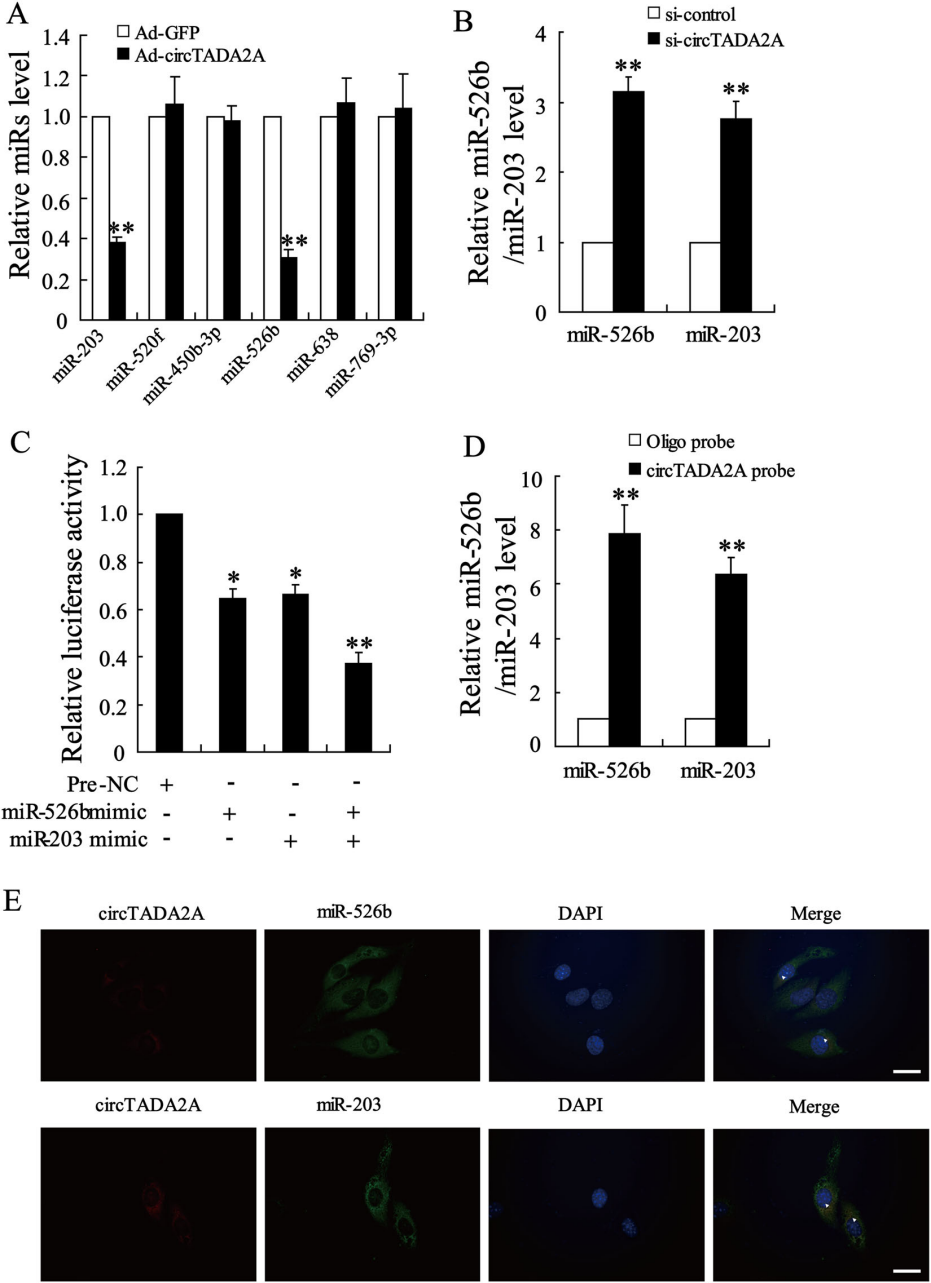
CircTADA2A bound to miR-203 and miR-526b. **a** LL-29 cells were transfected with Ad-circTADA2A or Ad-GFP, the expression levels of miR-203, miR-520f, miR-450b-3p, miR-526b, miR-638, and miR-769-3p were measured by qRT-PCR. **P < 0.01 vs Ad-GFP. **b** LL-24 cells were transfected with si-circTADA2A or si-control, the expression levels of miR-526b and miR-203 were measured by qRT-PCR. **P < 0.01 vs si-control. **c** HEK 293 T (293 T) cells were transfected with miR-526b mimic or miR-203 mimic or miR-526b mimic+miR-203 mimic or negative control (Pre-NC), relative luciferase activities of circTADA2A vector were measured using Dual-Luciferase Reporter Assay System. *P < 0.05, **P < 0.01 vs Pre-NC. **d** Detection of miR-526b and miR-203 using qRT-PCR in the samples pulled down by the circTADA2A probe and its negative control (Oligo probe). **P < 0.01 vs Oligo probe. **e** Colocalization between circTADA2A (red) and miR-526b/miR-203 (green) was observed (arrowheads) using FISH in LL-29 cells. The nuclei were stained with DAPI. Scale bar = 20 µm.

Studies have shown that circRNA could function as miRNA sponges, binding to miRNA directly and removing their suppressive effect on mRNA expression, thus participating in the regulation of gene expression^6^. Using a bioinformatics database (Circular RNA Interactome, https://circinteractome.nia.nih.gov/), we found several target miRNAs that had potential binding sites with circTADA2A. Among them, six miRNAs (miR-203, miR-520f, miR-450b-3p, miR-526b, miR-638, and miR-769-3p) with the highest prediction score were selected in our study. As shown in Fig. 3a, the overexpression of circTADA2A distinctly reduced expression levels of miR-203 and miR-526b while not affect expression levels of miR-520f, miR-450b-3p, miR-638, and miR-769-3p in human IPF fibroblastic cell line LL-29. Besides, compared with LL-24 cells, the expression levels of miR-526b and miR-203 were upregulated in LL-29 cells (Supplemental Fig. 2), indicating that miR-526b and miR-203 may take part in the development of IPF. Furthermore, in response to the transfection of si-circTADA2A, the expression levels of miR-203 and miR-526b were ascended in normal human fibroblastic cell line LL-24 (Fig. 2b). Next, Dual-luciferase Reporter Assay and RNA pull-down assay were performed to evaluate the endogenous combination of circTADA2A and miR-526b or miR-203. Compared with the negative control (Pre-NC), transfection of both miR-526b mimic and miR-203 mimic could decrease the luciferase activity of circTADA2A, and the luciferase activity presented further reduction after co-transfection of miR-526b mimic and miR-203 mimic (Fig. 3c). The RNA pull-down assay displayed that abundant miR-526b and miR-203 were observed in the complex pulled down by the circTADA2A probe than that of the Oligo probe (Fig. 3d). The results of the FISH assay further proved that circTADA2A and miR-526b/miR-203 were preferentially colocalized in the cytoplasm (Fig. 3e).

### CircTADA2A released Cav1 and Cav2 expressions via sponging miR-526b and miR-203

**Fig. 4:**
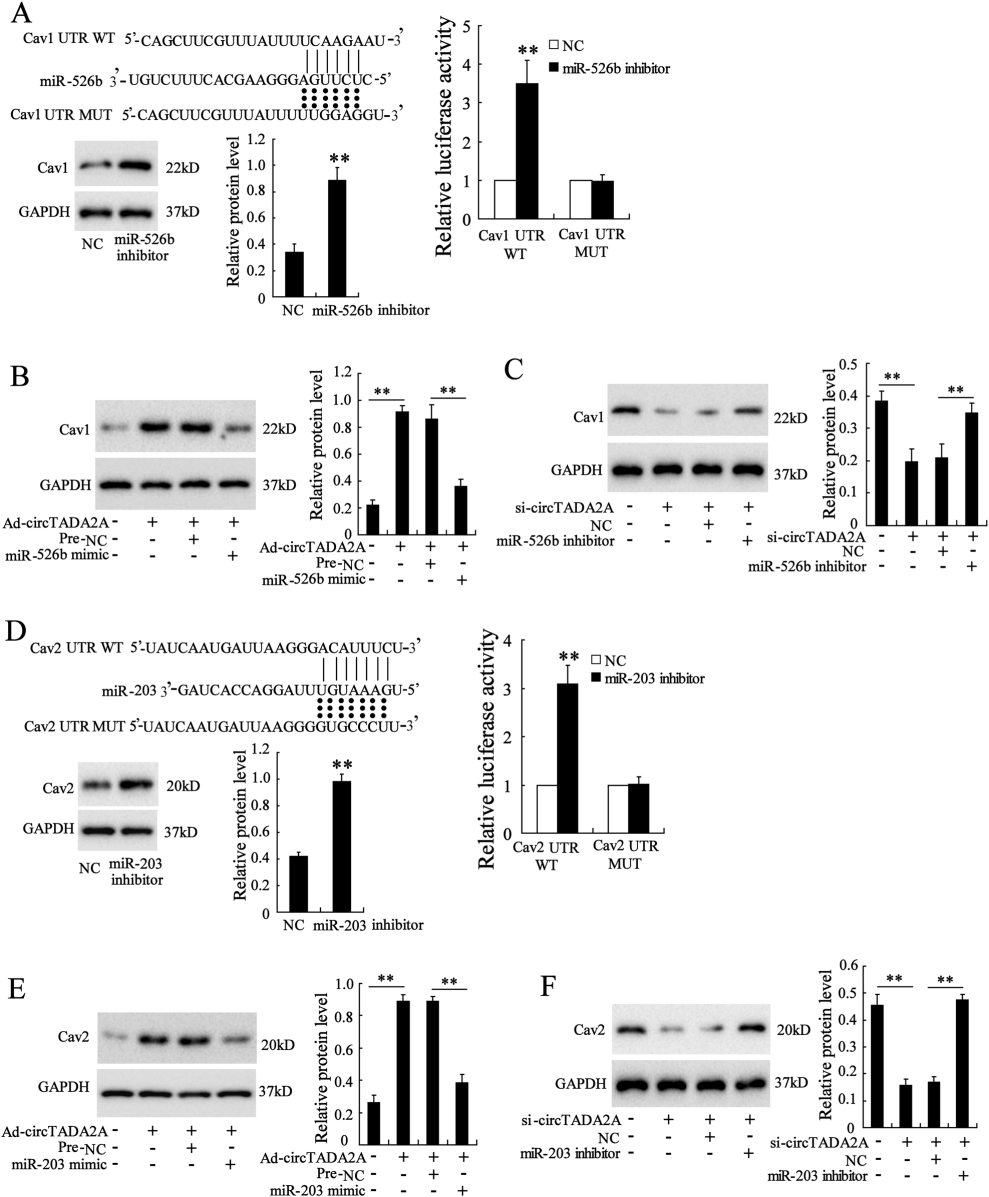
CircTADA2A released Cav1 and Cav2 expressions via targeting miR-526b and miR-203. **a** Putative binding sites between miR-526b and Cav1 were forecasted by Miranda. The expression of Cav1 was determined by western blot in LL-29 cells that have been transfected with miR-526b inhibitor or its negative control (NC). Luciferase activity of Cav1-3′-UTR wide type (WT) and Cav1-3′-UTR mutation (MUT) in 293 T cells that have been transfected with miR-526b inhibitor or NC. The expression of Cav1 was determined by western blot in **b** LL-29 cells that have been transfected with Ad-circTADA2A, miR-526b mimic, or the negative control of miR-526b mimic (Pre-NC), and **c** LL-24 cells that have been transfected with si-circTADA2A, miR-526b inhibitor, or NC. GAPDH was used as an internal control. **d** Putative binding sites between miR-203 and Cav2. The expression of Cav2 and the luciferase activity of Cav2-3′-UTR WT and Cav2-3′-UTR MUT were determined. The expression of Cav2 was determined by western blot in **e** LL-29 cells that have been transfected with Ad-circTADA2A, miR-203 mimic, or Pre-NC, and **f** LL-24 cells that have been transfected with si-circTADA2A, miR-203 inhibitor, or NC. GAPDH was used as an internal control. **P < 0.01 vs NC.

Cav1 and Cav2 are the major components of the caveolin protein family and expressed widely in lung tissues. It has been reported that Cav1 could suppress lung-fibroblasts activation by blocking the TGF-β signaling pathway^11^, Cav2 could decrease proliferation^12^ and apoptosis resistance^13^ of lung fibroblasts. What’s more, an online bioinformatics database (microRNA.org) revealed that miR-526b and miR-203 had the putative binding sites with Cav1 and Cav2, respectively. Firstly, the effect of miR-526b on Cav1 was evaluated. The miR-526b inhibitor raised Cav1 protein expression (Fig. 4a), and the Dual-luciferase reporter assays showed that compared with the negative control (NC), the luciferase activity of Cav1-3′-UTR wild-type (WT) was significantly upregulated in the cells transfected with miR-526b inhibitor. Whereafter, in IPF fibroblastic cell line (LL-29), the overexpression of miR-526b declined the high expression of Cav1, which was accelerated by Ad-circTADA2A (Fig. 4b) while not affect Cav2 (data not shown). In normal fibroblastic cell line (LL-24), the silence of miR-526b elevated the low expression of Cav1, which was suppressed by si-circTADA2A (Fig. 4c) while not affect Cav2 (data not shown). The above data indicating that circTADA2A upregulated Cav1 expression through sponging miR-526b. Afterward, following the same experimental procedure, we proved circTADA2A could also upregulate Cav2 expression through sponging miR-203 (Fig. 4d–f).

### The inhibitory effects of circTADA2A on lung-fibroblasts activation and proliferation were mediated via Cav1 and Cav2

**Fig. 5:**
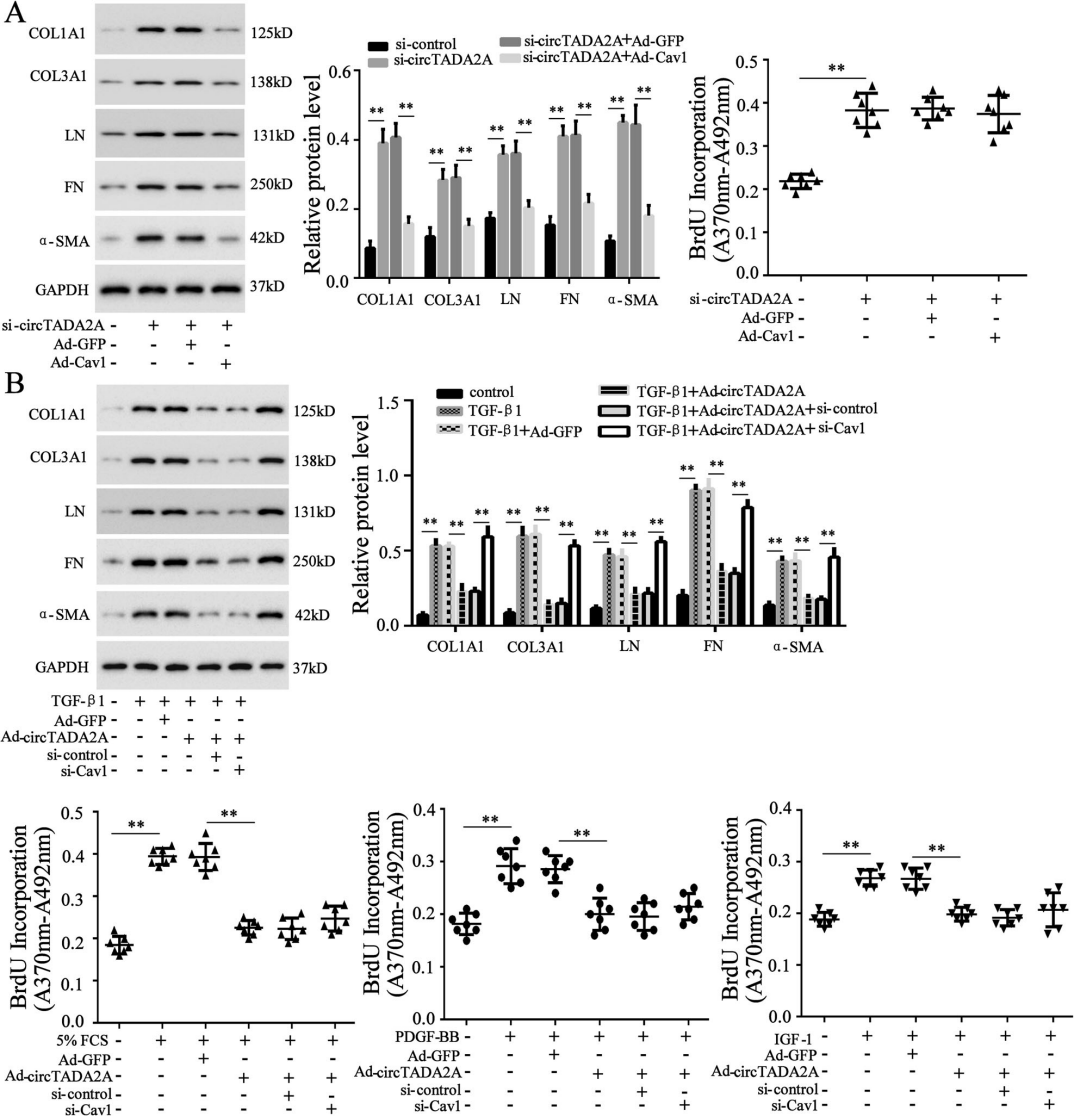
CircTADA2A inactivated lung fibroblasts via Cav1. LL-24 cells were transfected with **a** si-circTADA2A or Ad-Cav1+si-circTADA2A or Ad-GFP + si-circTADA2A or **b** Ad-circTADA2A or Ad-GFP or Ad-circTADA2A+si-control or Ad-circTADA2A+si-Cav1, and then stimulation with 5% FCS or 60 ng/ml PDGF-BB or 200 ng/ml IGF-1 or 10 ng/ml TGF-β1 for 6 h. The expression of COL1A1, COL3A1, LN, FN, and α-SMA were measured by western blot, and the cell proliferation was measured by BrdU incorporation assay. **P < 0.01.

**Fig. 6:**
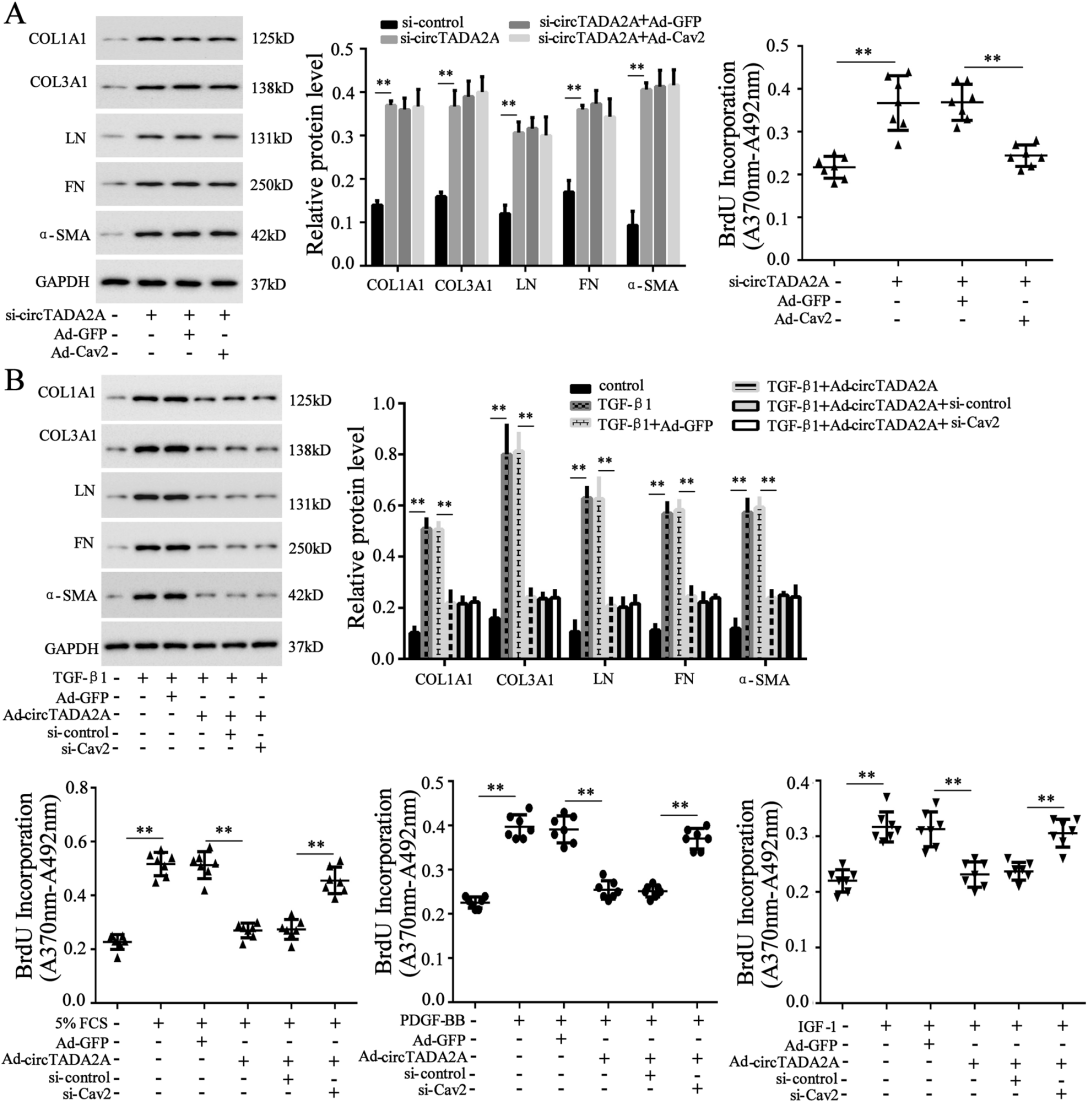
CircTADA2A inhibited lung-fibroblasts proliferation via Cav2. LL-24 cells were transfected with **a** si-circTADA2A or si-circTADA2A+Ad-Cav2 or si-circTADA2A+Ad-GFP or **b** Ad-circTADA2A or Ad-GFP or Ad-circTADA2A+si-control or Ad-circTADA2A+si-Cav2, and then stimulation with 5% FCS or 60 ng/ml PDGF-BB or 200 ng/ml IGF-1 or 10 ng/ml TGF-β1 for 6 h. The expression of COL1A1, COL3A1, LN, FN, and α-SMA were measured by western blot, and the cell proliferation was measured by BrdU incorporation assay. **P < 0.01.

Once demonstrated that circTADA2A could release the expressions of Cav1 and Cav2 via acting as sponges of miR-526b and miR-203, we decided to confirm whether circTADA2A restrained lung-fibroblasts proliferation and activation through Cav1 and Cav2. Firstly, in LL-24 cells that have been transfected with si-circTADA2A, the overexpression of Cav1 reversed the promotion effect of si-circTADA2A, decreased the lung-fibroblasts activation and ECM production (Fig. 5a), while the overexpression of Cav2 reduced lung-fibroblasts proliferation induced by si-circTADA2A (Fig. 6a), hinting that circTADA2A inactivated lung fibroblasts via Cav1 and reduced lung-fibroblasts proliferation via Cav2. To further verify this finding, TGF-β1 was added in the culture medium of LL-24 cells to induce cell activation, while FCS, PDGF-BB, and IGF-1 were added to induce cell proliferation. The overexpression of circTADA2A restrained LL-24 cells from activation and proliferation, on the contrary, the silence of Cav1 eliminated the inhibitory effect of Ad-circTADA2A on lung-fibroblasts activation (Fig. 5b), and the silence of Cav2 reversed the inhibitory effect of Ad-circTADA2A on cell proliferation (Fig. 6b).

### CircTADA2A alleviated fibrogenic responses in lung-fibrosis mouse

**Fig. 7:**
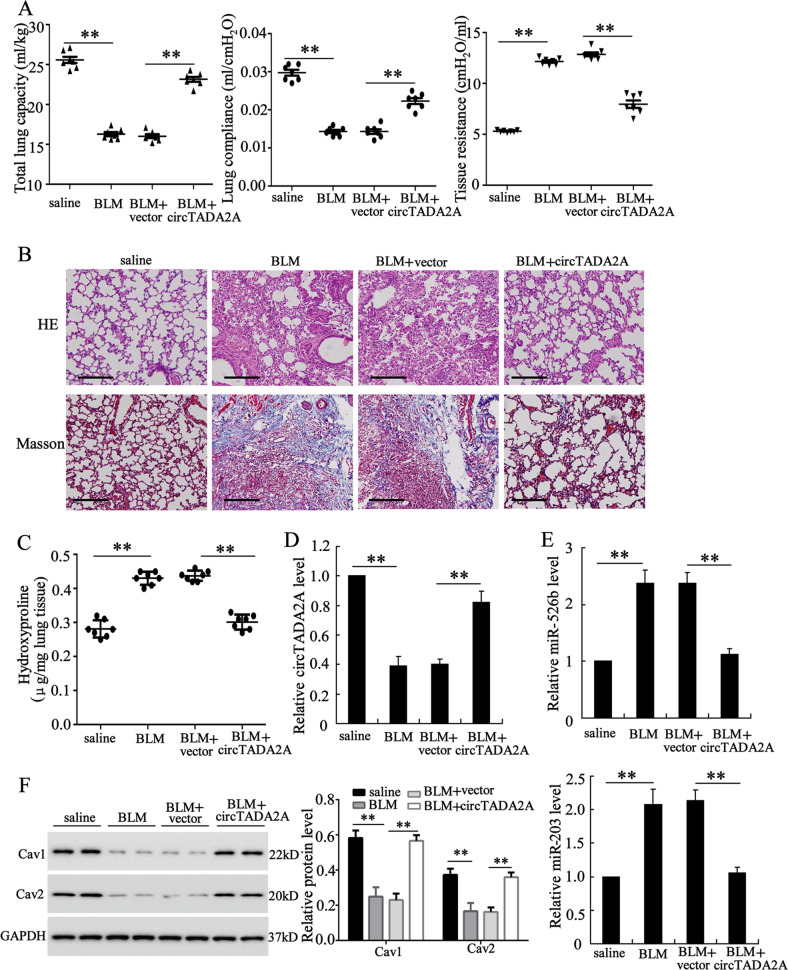
CircTADA2A alleviated pulmonary fibrosis in lung-fibrosis mice. Mice were divided into four groups: saline (*n* = 7), bleomycin (BLM; *n* = 7), BLM + adenovirus circTADA2A (circTADA2A; *n* = 7), and BLM + adenovirus vector (vector; *n* =7). Mice were infected with Ad-circTADA2A or Ad-vector, 2 days later, BLM (3 U/kg) in 50 μl saline was administered intratracheally in mice to induce pulmonary fibrosis. **a** Two weeks after BLM stimulation, lung-function measurements of mice (total lung capacity, lung compliance, and tissue resistance) were measured, and then the mice were sacrificed and the lung tissues were collected for the following experiments. **b** H&E and Masson staining of each group. Scale bars = 200 μm. **c** Hydroxyproline content of lung homogenates was evaluated using a Hydroxyproline Assay Kit. **d** The expression level of circTADA2A was measured by qRT-PCR. **e** The expression levels of miR-526b and miR-203 were measured by qRT-PCR. **f** The expression levels of Cav1 and Cav2 were measured by western blot. GAPDH was used as an internal control. **P < 0.01.

To assess the potential therapeutic effect of circTADA2A in vivo, mice were infected with Ad-circTADA2A or negative control (vector) before the establishment of a lung-fibrosis mouse model induced by BLM, and the qRT-PCR results proved the infections worked (Fig. 7d). We observed that the lung function of circTADA2A-treated lung-fibrosis mice was improved as compared to vector-treated lung-fibrosis mice (Fig. 7a). In line with the lung-function analysis, analysis of lung sections manifested Ad-circTADA2A could mainly preserve the alveolar structure and greatly reduce collagen deposition in comparison with vector-treated lung-fibrosis mice (Fig. 7b). Meanwhile, in circTADA2A-overexpressed lung-fibrosis mice, the hydroxyproline content was lower than that of vector-treated lung-fibrosis mice (Fig. 7c). Additionally, Ad-circTADA2A was also able to downregulated the BLM-induced high expression levels of miR-256b and miR-203 (Fig. 7e) and upregulated the BLM-induced low protein levels of Cav1 and Cav2 (Fig. 7f). The above data signified that miR-256b/Cav1 and miR-203/Cav2 also took part in the therapeutic effect of circTADA2A on lung-fibrosis mice.

## Discussion

The etiology of IPF has been explained by several hypotheses, among them, the accumulation of ECM induced by hyperproliferating and activating fibroblasts are considered to be the core mechanism. The present study, performed in primary human lung fibroblasts (isolated from healthy and IPF lung tissues), lung-fibroblasts cell lines and a mouse model of lung fibrosis, identified new regulatory pathways concerning fibroblasts activation and proliferation, involving circTADA2A, miR-526b, miR-203, Cav1, and Cav2. Briefly, we clarified that high expression of circTADA2A suppressed fibroblasts activation by upregulating Cav1 via targeting miR-526b, reduced fibroblasts proliferation by upregulating Cav2 via targeting miR-203, thus decreasing ECM deposition and alleviating IPF. Our research forwarded strong evidence for the vital role of circTADA2A in the progression of IPF, providing novel perspectives for IPF treatment.

Whole-transcriptome RNAs sequencing revealed the existence of noncoding RNAs (ncRNAs). In recent years, dysfunction of ncRNAs has been proved to be associated with various complex human diseases, and increasing researchers paid attention to the roles of ncRNAs in IPF. Savary et al.^14^ identified long noncoding RNA (lncRNA) DNM3OS as a critical downstream effector of TGF-β during the activation of lung fibroblasts. Yang et al.^15^ showed that miR-200 family members (200a, 200b, and 200c) reversed fibrogenic responses in IPF via restraining epithelial-mesenchymal transition (EMT). Nevertheless, to date, compared with lncRNA and miRNA, little is known about the role of circRNA in IPF. Through a circRNA microarray, Li et al.^5^ screened out 67 circRNA presented visibly dysregulated in the plasma of IPF patients, implying the potential role of circRNA in IPF, while the explicit regulatory mechanism of circRNA in IPF is confusing. In our study, we observed a distinct downregulation of circTADA2A (hsa_circ_0043278) in the primary human lung fibroblasts isolated from IPF lung tissues. Then, we confirmed this reduction of circTADA2A in both human IPF fibroblastic cell lines and the activation process of a human normal fibroblastic cell line (Fig. 1). Further study showed that the overexpression of circTADA2A effectively suppressed the proliferation and activation of lung fibroblasts driven by fibrogenic growth factors (Fig. 2) and expounded the specific mechanism. The study herein investigated the specific role of circRNA in IPF and clearly point out the anti-fibrosis effect of circTADA2A.

CircTADA2A is identified from exons 5 and 6 of the Transcriptional Adaptor 2A (TADA2A) gene, and its role in disease or biological process is rarely reported. In the osteosarcoma cell line, Wu et al.^16^ determined that circTADA2A was mainly located in the cytoplasm and exhibited marked stability under RNase R treatment, revealing the potential of circTADA2A on function as miRNA sponges and abolish the downstream effects of these miRNAs on target mRNAs. In our study, through luciferase reporter gene experiments and RNA pull-down assay, we confirmed circTADA2A could act as sponges of miR-526b and miR-203, hinting that except IPF, circTADA2A may take part in the regulatory network of more human diseases related to miR-526b or miR-203. Chi et al.^17^ found that miR-203 inhibited cell proliferation, invasion, and migration of non-small-cell lung cancer by targeting RGS17. Interestingly, in our study, we found that miR-203 negatively regulated Cav2 expression, which could inhibit the proliferation of fibroblasts, hinting that miR-203 may promote cell proliferation in lung fibroblasts. In line with our study, Ren et al.^18^ also found that miR-203 promoted proliferation in pancreatic cancer cells via degrading salt-inducible kinase 1. As reported, miRNA can bind to the 3′UTR region of target mRNA and represses gene expression^19^. Therefore, the function of the miRNA is determined by its downstream target gene. In different cell types or different disease states, miRNA could target different target genes and exhibited different functions. Besides, the researches of miR-526b were focused on its anticancer effect^20^, in the present study, we firstly reported miR-526b was related to the activation of lung fibroblasts by targeting Cav1.

Caveolin protein family is the major protein component of caveolae (invaginations of the plasma membrane) and takes part in the modulation effect of caveolae on signal transduction^21^. Three numbers of caveolin protein family have been identified: caveolin-1, -2, and -3. Among them, Cav1 is expressed widely in lung tissues and exhibits an inhibitory effect on fibroblasts activation via directly interacting with the TGF-β1 receptor (TGF-β1R) and inhibiting the excessive expression of TGF-β1^22^. TGF-β1 is an essential prompter of collagen amassing and fibrogenesis, driving fibroblasts activation via TGF-β/Smad2/3 and PI3K/Akt signaling^23,24^. An evident decline of Cav1 expression in lung tissues of IPF patients has been observed^25^ and the low expression of Cav1 resulted in the activation of fibroblasts^11^. In line with this, in our study, Cav1 displayed low expression in IPF fibroblastic cell line (Fig. 4b), and the dual-luciferase reporter gene assay showed that the decline was due to the combination of miR-526b and 3′ UTR of Cav1, the overexpression of circTADA2A released Cav1 expression via sponging miR-526b. What’ more, the silence of Cav1 reversed the inhibitory effect of Ad-circTADA2A on lung fibroblasts induced by TGF-β1 (Fig. 5b), confirming that circTADA2A repressed TGF-β1-induced fibroblasts activation via Cav1. Except Cav1, emerging evidence showed that Cav2 was also involved in the regulation of pulmonary fibrosis. Almeida et al.^12^ reported that during the pulmonary fibrosis induced by bleomycin, compared with wild-type mice, the TGF-β level was not altered while the proliferation of cells was promoted in the lung tissues of Cav2^−/−^ mice. In the present study, we found that the overexpression of Cav2 reversed the promotion effect of si-circTADA2A on cell proliferation while not affect fibroblasts activation (Fig. 6a), confirming that Cav2 was only responsible for the inhibitory effect of circTADA2A on fibroblasts proliferation.

In conclusion, the current study elucidated that circTADA2A could repress fibroblasts activation and proliferation via miR-526b/Cav1 and miR-203/Cav2 pathway, thus alleviating IPF. Our study clarified the anti-fibrosis effect of circTADA2A for the first time and providing new perspectives for IPF therapy and circRNA investigation.

## Supplementary information

Supplementary Information

Supplementary Information 2

Supplementary Information 3

Supplementary Information 4
