# Supplementary material for: CircRNA TADA2A relieves idiopathic pulmonary fibrosis by inhibiting proliferation and activation of fibroblasts
Source: Cell Death Dis. 2020 Jul 21;11(7):553. doi: 10.1038/s41419-020-02747-9 (PMC7374112; doi:10.1038/s41419-020-02747-9)
Supplement: Supplementary file 1 — Supplementary Information [file 41419_2020_2747_MOESM1_ESM.docx]

**Supplemental Figure 1** LL-29 cells were transfected with Ad-GFP or Ad-circTADA2A. Forty-eight hours later, the expressions of COL1A1, COL3A1, LN, FN, and α-SMA were measured by western blot. GAPDH was used as an internal control.
