# Supplementary material for: CircRNA TADA2A relieves idiopathic pulmonary fibrosis by inhibiting proliferation and activation of fibroblasts
Source: Cell Death Dis. 2020 Jul 21;11(7):553. doi: 10.1038/s41419-020-02747-9 (PMC7374112; doi:10.1038/s41419-020-02747-9)
Supplement: Supplementary file 2 — Supplementary Information 2 [file 41419_2020_2747_MOESM2_ESM.docx]

**Supplemental Figure 2** The expression levels of miR-526b and miR-203 were measured in LL-24 and LL-29 cells using qRT-PCR. ***P*<0.01 vs LL-24 cells.
