# Supplementary figures and images for: CircRNA TADA2A relieves idiopathic pulmonary fibrosis by inhibiting proliferation and activation of fibroblasts
Source: Cell Death Dis. 2020 Jul 21;11(7):553. doi: 10.1038/s41419-020-02747-9 (PMC7374112; doi:10.1038/s41419-020-02747-9)

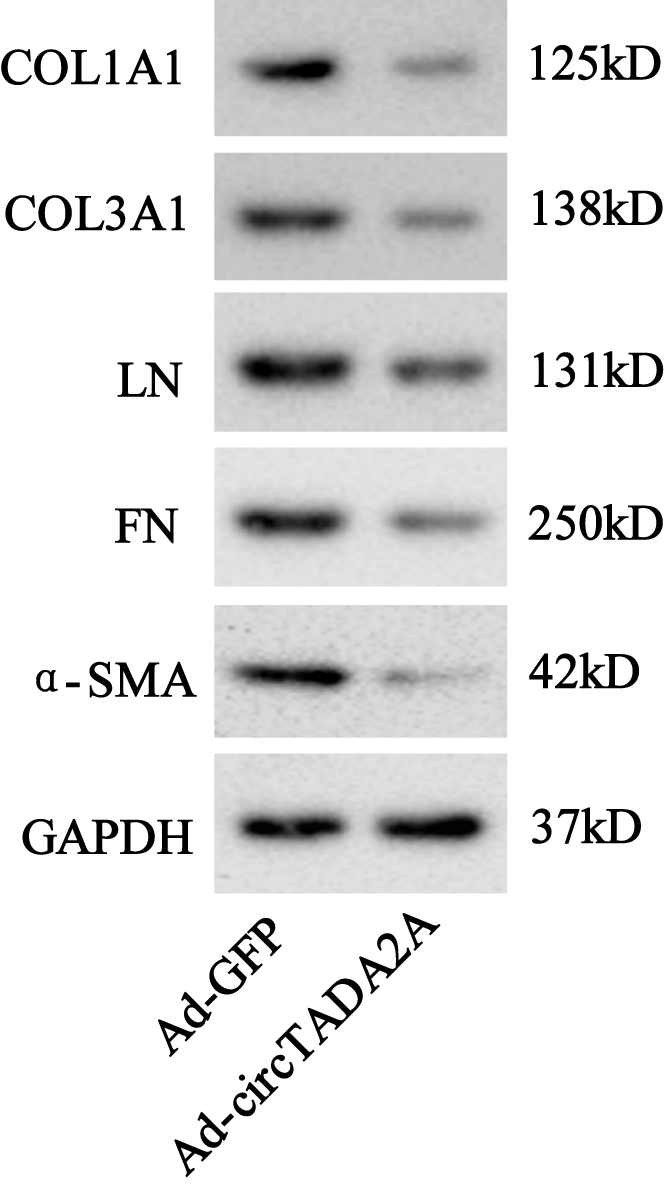

Supplement: Supplementary file 3 — Supplementary Information 3 [file 41419_2020_2747_MOESM3_ESM.tif]

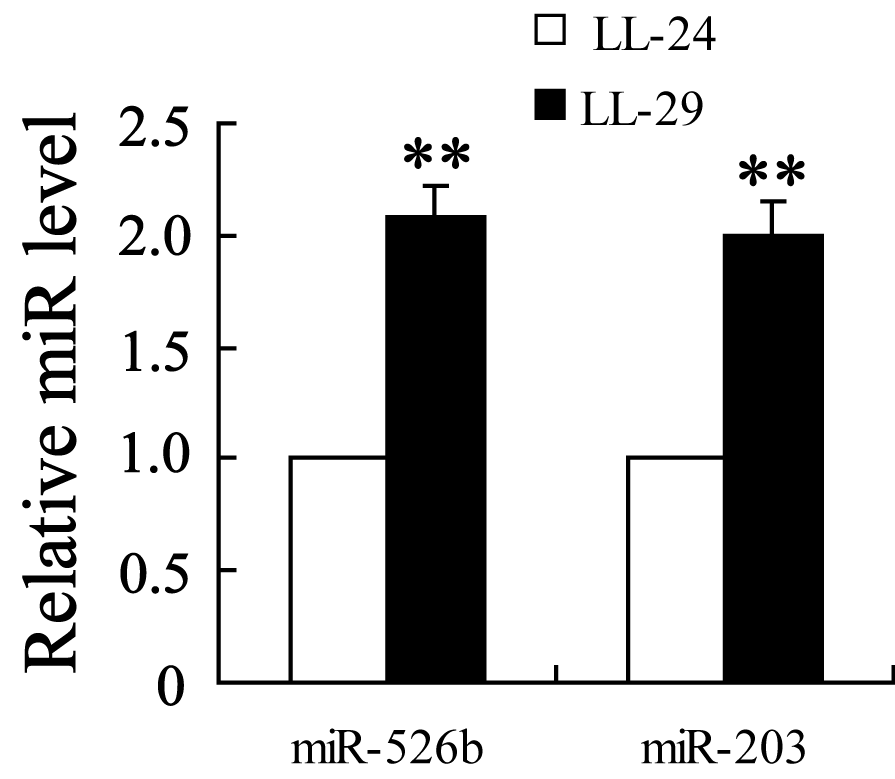

Supplement: Supplementary file 4 — Supplementary Information 4 [file 41419_2020_2747_MOESM4_ESM.tif]
